# Chlorhexidine for prevention of alveolar osteitis: a randomised clinical trial

**DOI:** 10.1590/1678-7757-2017-0245

**Published:** 2018-04-18

**Authors:** Diego Halabi, Jose Escobar, Cyntia Alvarado, Nicolette Martinez, Carlos Muñoz

**Affiliations:** 1 Universidad Austral de Chile, Facultad de Medicina, Escuela de Odontologia, Valdivia, Chile; 2 Servicio de Salud Valdivia, Valdivia, Chile

**Keywords:** Preventive dentistry, Oral surgery, Tooth extraction, Chlorhexidine, Clinical trial, Alveolar osteitis

## Abstract

**Objective:**

To determine the effectiveness of chlorhexidine 0.12% mouthwash (CHX) after tooth extraction for the prevention of alveolar osteitis (AO).

**Material and methods:**

We conducted a double-blind randomised clinical trial stratified by risk factors. We enrolled a cohort of 822 patients who underwent dental extractions, and were considered to be at risk of developing AO (previous surgical site infection, traumatic extraction, and tobacco smoking). After extraction, patients were randomly allocated for CHX group or placebo group, matched by risk factors. The primary outcome was clinical diagnosis of AO: increasing postoperative pain for 4 d within and around the socket, and total or partial breakdown of the blood clot in the socket with or without bone exposure.

**Results:**

Follow-up was completed by 744 participants (372 chlorhexidine and 372 placebo). We detected no significant differences between the two groups at baseline. After completed follow-up, risk factors were equally distributed between the two groups. Overall incidence of OA was 4.97%, in which 27 participants treated with placebo (7.26%) and 10 participants treated with CHX (2.69%) developed AO. CHX reduced the incidence of AO by 63% [Absolute Risk Reduction: 4.57 (95% CI 1.5-7.7), Number Needed to Treat: 21.88 (95% CI 13.0-69.3), Fisher's exact test: p=0.006]. No adverse effects were reported.

**Conclusion:**

The use of chlorhexidine 0.12% mouthwash after tooth extraction is safe and effective in reducing the incidence of AO in high-risk patients.

## Introduction

Patients who undergo dental extractions are at risk of developing post-operative complications, and the most common is the alveolar osteitis (AO)^12^. Our group^8^ recently reported an incidence of 6.4% of AO, and we determined by a logistic regression model that previous surgical site infection, tobacco smoking and traumatic extraction are risk factors for developing AO. These risk factors explain why there were changes in prevalence ranging from 3.9% up to 29.6% for the third molar^10^ ^,^ ^23^.

With a risk model that predicts the development of AO, it is possible to implement preventive health care to those individuals at high risk. Thus, clinicians can help their patients to avoid the severe pain caused by this complication^17^, which traditionally receives symptomatic treatment of uncertain effectiveness^16^. Hence, clinicians can reduce health care costs and provide comfort for patients^9^.

It has been proposed that chlorhexidine 0.12% mouthwash can be used after extraction for the prevention of AO^10^ ^,^ ^13^ ^,^ ^18^. Chlorhexidine is the most widely used antiseptic in dentistry because its broad-spectrum antibacterial effectiveness is well established^2^, so it can be implemented as a simple and inexpensively public health policy.

However, the clinical trials that support chlorhexidine 0.12% mouthwash are inconclusive, showing methodological weaknesses and having a high risk of bias^5^ ^,^ ^9^ ^,^ ^24^. Therefore, the need to conduct randomized clinical trials of better quality and including risk factors is imperative.

Here, we conducted a double-blind randomised clinical trial stratified by risk factors to determine the effectiveness of CHX after tooth extraction for the prevention of AO.

## Material and methods

### Trial design

We conducted a randomized, double-blind, parallel-group, stratified by risk factors, placebo-controlled, clinical trial in two public community dental clinics in Valdivia, Chile (population 154,559). All participants agreed to participate by signing an informed consent form, according to the recommendations of the Declaration of Helsinki. The protocol of this study was approved by the Research Ethics Committee of the Public Health Service of Valdivia. The trial was registered as ISRCTN14646628.

### Population

We recruited patients registered to receive dental care from a list of random numbers generated by computer, from April 2013 to December 2015. Inclusion criteria were adults of 18 years or older with clinical indications for tooth extraction, and who presented at least one of the following risk factors for developing AO: tobacco smoker (consumption of ≥5 cigarettes 24 h before extraction), previous surgical site infection (clinical diagnosis of chronic periodontitis, acute periodontal conditions, apical periodontitis, pericoronitis, fungal infections, or dental pulp gangrene) and/or traumatic extraction (lifting a flap, use of elevators for >4 min, and/or rotary instruments).

Exclusion criteria were patients requiring extraction in the operating theater, residents of rural areas who manifested difficulty in returning for follow-up, patients allergic to chlorhexidine, patients under antimicrobial therapy, antibiotic prophylaxis, or antibiotics therapy after extraction.

Dental extractions were performed by dental surgery team from the emergency department of the clinic, in accordance with standard procedures as defined by the National Health Service^14^.

### Interventions

After surgery, patients were allocated to the treatment group or the placebo group. Treatment consisted of a mouthwash with 15 ml chlorhexidine 0.12% (Oralgene^®^ Mouthwash 0.12%, Maver, Chile) for 30 s, twice a day for 7 d, starting 24 h after extraction. The placebo was sterile water, with the same indications for use. Both chlorhexidine and placebo were stored in similar brown plastic bottles, and instructions were given orally and in writing to each participant.

To guarantee that in both groups (treatment and placebo) the risk of alveolar osteitis was similar and comparable, the assignment was performed by randomization, stratified by risk factors, and 7 groups were formed with the following possible combinations: smoker; previous infection; traumatic extraction; smoker + previous infection; smoker + traumatic extraction; previous infection + traumatic extraction; smoker + previous infection + traumatic extraction. To avoid the risk of having more patients in a group, we stored black envelopes in a box containing a paper with the letter C for chlorhexidine or P for placebo (half of each). The envelopes were chosen for each patient after the extraction and transported to another room (without opening them); they were read only by one of the authors, who then distributed the chlorhexidine or placebo accordingly. For each patient who was assigned to a group, the subsequent patient who arrived with the same risk factors was matched to the opposite group, and the respective envelope was discarded (to ensure homogeneity of groups).

### Outcome measures

We recorded age (years), gender (male or female), tooth location (mandibular or maxillary), diagnosis or previous surgical site infection (yes or no, as described), smoking (smoker or non-smoker, as described) and traumatic extraction (yes or no, as described) before tooth extraction for each patient.

The primary outcome was positive diagnosis of AO one week after tooth extraction. Positive diagnosis of AO was identified by the authors in patients with the following characteristics: 1) increasing postoperative pain intensity for 4 d within and around the socket and 2) total or partial breakdown of the blood clot in the socket with or without bone exposure.

At the same time, we assessed hypersensitivity to chlorhexidine (contact dermatitis, pruritus, vesicle formation, urticaria, dyspnea, or anaphylactic shock), dysgeusia (alteration of taste perception, bitter taste or burning) or pigmentation (staining of teeth and/or tongue) as potentially adverse events.

We treated patients who developed alveolar osteitis and other complications in accordance with the available clinical protocols of the Chilean Health Ministry^14^.

### Sample size

We estimated sample size using data published previously by Halabi, et al.^8^ (2012), expecting an incidence reduction of two-thirds. The *P_A_* expected incidence of disease (AO) in the placebo group was 6.14%, while the *P_B_* expected incidence of disease (AO) in the CHX group was 2.05%. Additionally, *k* groups ratio of sample sizes between groups was 1:1. The power of the study was set at 80% (β=0.20), with α = 0.05 as the significance level. Based on these parameters, we applied the following equation^19^:

$$sample\;size=\left(\frac{p_{A}(1-p_{A})}{k}+p_{B}(1-p_{B})\right){\left(\frac{z_{1}-\frac{\alpha }{2}+z_{1}-\beta }{p_{A}-p_{B}}\right)}^{2}\text{.}$$

### Statistics

We performed the statistical analysis by DH using R 3.3.1 (R Foundation for Statistical Computing, Vienna, Austria). We used the Fisher's exact test to detect significant differences in the incidence of AO between CHX and placebo groups, and also to analyse gender, tooth location, patient smoking, previous infection and traumatic extraction variables. We used unpaired t-test was to detect significant differences in age between CHX and placebo groups. We determined the incidence of AO for both groups and calculated the Number Needed to Treat (NNT). For all tests, statistical differences were determined to be significant at p<0.05.

## Results

**Figure 1:**
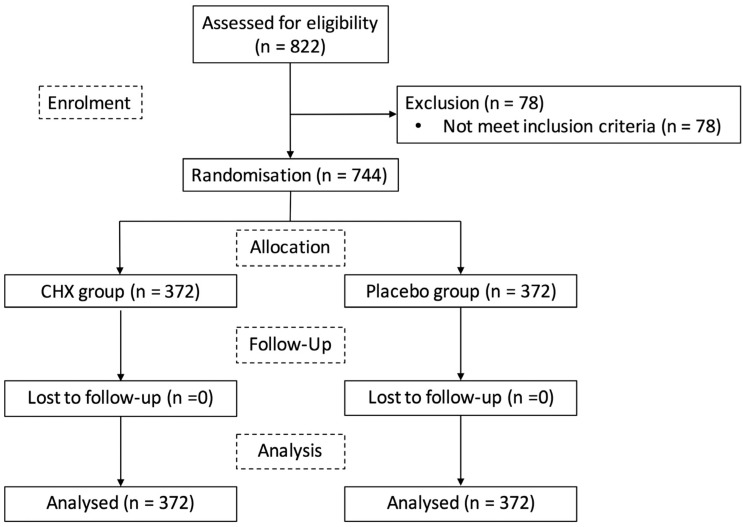
CONSORT flow diagram of patients included in the final analysis

From April 2013 to December 2015, we recruited 822 participants. Out of these, 744 met inclusion criteria and completed the follow-up. They were allocated into two groups of 372 for treatment of chlorhexidine 0.12% or placebo. Figure 1 shows the flow diagram of participants.

As seen in Table 1, we included 381 female participants and 363 males at baseline. The mean age was 43.43 years (SD 14.99). Comparison of baseline data between the group treated with chlorhexidine 0.12% and the one treated with placebo did not show statistically significant differences for age [t(degrees of freedom)=0.917 (742), p=0.359], gender (p=0.463), location of the extracted tooth (p=0.238), previous infection in surgical site (p=0.999), tobacco smoke (p=0.999) or traumatic extraction (p=0.999).

**Table 1:** Baseline data of participants, mean age (SD), and frequency (%) of gender, location of the tooth extracted, previous surgical site infection, tobacco smoke and traumatic extraction

	Chlorhexidine (n=372)	Placebo (n=372)	Total (n=744)	P value
Age (years ± SD)	43.93±15.15	42.92±14.84	43.43±14.99	0.396
Gender [n (%)]				
Female	185 (48.6%)	196 (51.4%)	381 (100%)	0.463
Male	187 (51.5%)	176 (48.5%)	363 (100%)	
Tooth location [n (%)]				
Mandibular	158 (47.5%)	175 (52.5%)	333 (100%)	0.238
Maxillary	214 (52.1%)	197 (47.9%)	411 (100%)	
Previous surgical site infection [n (%)]				
Yes	339 (50%)	339 (50%)	678 (100%)	0.999
No	33 (50%)	33 (50%)	66 (100%)	
Smoking [n (%)]				
Smoker	152 (49.8%)	153 (50.2%)	305 (100%)	0.999
Non-smoker	220 (50.1%)	219 (49.9%)	439 (100%)	
Traumatic extraction [n (%)]				
Yes	51 (50%)	51 (50%)	102 (100%)	0.999
No	321 (50%)	321 (50%)	642 (100%)	

Once all participants completed the follow-up, we observed that the risk factors were equally distributed between the two groups, without statistically significant differences (see details in Table 2).

**Table 2:** Frequency of patients by matched risk factors for alveolar osteitis, distributed by treatment group

Risk factor	Chlorhexidine (n=372)	Placebo (n=372)	Total (n=744)
S	24	24	48
PI	191	190	381
TE	3	3	6
S+PI	106	107	213
S+TE	6	6	12
PI+TE	26	26	52
S+PI+TE	16	16	32
Total	372	372	744

S: Tobacco smoke

PI: Previous infection in surgical site

TE: Traumatic extraction

We diagnosed 37 cases of AO, with an overall prevalence of 4.97%. In the group treated with chlorhexidine 0.12% mouthwash we diagnosed 10 participants (2.69%) with AO, while in the placebo group they were 27 (7.26%) (p=0.006, statistical power=0.821). CHX reduced the incidence of AO by 63% [Absolute Risk Reduction: 4.57 (CI95% 1.5-7.7), Number Needed to Treat: 21.88 (CI95% 13.0-69.3)].

See more details in Table 3.

**Table 3:** Incidence of alveolar osteitis in patients treated with chlorhexidine 0.12% mouthwash or placebo

	AO	Health	Total	ARR (95% CI)	NNT (95% CI)	P value
Chlorhexidine	10	362	372	4.57%	21.88	0.006*
Placebo	27	345	372	(1.5 – 7.7)	(13.0 – 69.3)	
Total	37	437	744			

AO: Alveolar Osteitis; ARR: Absolute Risk Reduction; NNT: Number Needed to Treat; CI: Confidence interval;

* p<0.05; power=0.822 (Fisher's exact test)

No patient had hypersensitivity to chlorhexidine, dysgeusia or tooth pigmentation.

## Discussion

We conducted a randomised, controlled trial to assess the effectiveness of postoperative chlorhexidine 0.12% mouthwash to reduce the prevalence of postextraction AO, and we contrasted it with a placebocontrolled group. We presented new findings in which chlorhexidine treatment reduces the incidence of AO by 63% in high-risk patients, with strong statistical power.

Subgroup analysis showed no difference in the incidence of AO and location of the extracted tooth. Mandibular teeth developed the same rate of AO as that of the maxillary teeth, and anterior teeth showed a similar prevalence to posterior teeth. These findings are consistent with our previous observations^8^. Therefore, our results can be extrapolated to any patient who requires an extraction, regardless of the location of the tooth.

AO incidence in this study was quite similar to that which we reported previously: 4.97% *versus* 6.4%^8^. We suspect that the incidence might have been higher in the placebo group on this occasion (incidence was 7.26%), since the study sample consisted of patients with an increased risk of developing alveolar osteitis. However, we can explain this because the placebo effect can work as an effective treatment, especially in conditions associated with pain^15^ ^,^ ^22^.

Our results have some similarities with those reported in the literature with similar interventions. Ragno and Szutnik^18^ (1991) reported a statistically significant reduction of 50% in the prevalence of AO after the extraction of mandibular third molars [risk ratio (RR) 0.5; 95% confidence interval (CI), 0.27 to 0.93]. Larsen^13^ (1991) studied the AO preventive effect of chlorhexidine 0.12% mouthwash after mandibular third molar removal and observed a reduction in the prevalence of 60% (RR 0.4; 95% CI, 0.21 to 0.75). Hermesch, et al.^10^ (1998) reported that chlorhexidine 0.12% mouthwash reduced to 38% the prevalence of AO after extraction of impacted mandibular third molars (RR 0.62; 95% CI, 0.40 to 0.96). In contrast, Delilbasi, et al.^7^ (2002) found that 0.2% chlorhexidine had no statistically significant effect in reducing the prevalence of AO after mandibular third molar removal (RR 0.88; 95% CI, 0.45 to 1.72). However, these studies have a high risk of bias^6^ ^,^ ^9^ ^,^ ^24^.

Our study has several strengths compared to those reported in the literature. To minimize selection bias, we randomly included only the patients who had the risk factors that we reported previously^8^. Thus, the preventive effect was studied in a group known to have a high risk of developing AO, and not in persons who have not had chances to develop AO, regardless of receiving treatment or not.

We assigned participants to each group randomly, blindly and matched by risk factors. Thus, both groups had similar distribution in the risk of developing AO, and the results cannot be taken by a simple imbalance in the risk to develop AO in any of the groups.

Additionally, we did not observe losses regarding follow-up or treatment withdrawals, minimising attrition bias risk. This was because we designed a protocol of four phone calls encouraging the patient to attend the clinical control, and our local community clinics allow to “retain” patients.

Studies that we compared previously used a model of impacted mandibular third molars. We consider that this model does not differ from ours, in which we included the extraction of any tooth in the mouth. The only difference is that in the extraction of impacted third molars, the risk factor “traumatic extraction” is exacerbated, which explains why the prevalence of AO in these studies is higher. In addition, we controlled the tooth location, which gives us the advantage that our results can be extrapolated to a wider population, and not be limited to only those procedures with a high level of tissue damage (*i.e.* mandibular third molar extraction).

Nonetheless, this study has some weaknesses. Firstly, it is difficult to ensure that patients have followed the full treatment, because they administered themselves in their homes. To reduce the risk of poor treatment procedure, we phoned the patients once a day for the first 4 d to remind them to use the rinse. Additionally, we required them to bring the bottles of mouthwash to the dental control, to ensure that it was fully used. If this had not been the case, the patient would have been discarded from the follow-up. We used sterile water as placebo, which could have been a risk if the patient recognized it as such, but in the reinforcement phone calls, we did not detect this situation in any patient. Secondly, the operational definition of smokers was changed in relation to our previous study^8^ *(i.e.* consumption of ≥5 cigarettes 24 h after extraction, to consumption of ≥5 cigarettes 24 h before extraction). To include the patient as a smoker we had to know if they smoked previously.

It has been proposed that intra-alveolar application of chlorhexidine 0.2% gel may be an effective treatment to prevent AO^11^ ^,^ ^20^ ^,^ ^21^. However, the evidence supporting this preventive therapy has the same inconsistencies and weaknesses as the studies of chlorhexidine 0.12% mouthwash^6^. There is no biological plausibility that this form of treatment can be more effective, because a concentration of 0.2% shows no additional antibacterial benefits than the concentration of 0.12%^3^, and the substantivity of the chlorhexidine mouthwash is sufficient to maintain its effect for 12 h^4^. There is no evidence that chlorhexidine may have a negative effect on haemostasis, and the mode of use as a mouthwash does not interfere with clot formation, since our application protocol begins 24 h after the extraction, when the clot has already been stabilized and begins to be replaced with granulation tissue^1^.

With the strengths and weaknesses of our study, we conclude that the use of chlorhexidine 0.12% mouthwash after tooth extraction is highly effective compared with placebo mouthwash in preventing AO in high-risk patients. Clinical trials evaluating preventive effects should consider the risk factors in the study design to minimise risk of bias.
